# Clinical and genetic features of a case with juvenile onset sandhoff disease

**DOI:** 10.1186/s12883-023-03267-7

**Published:** 2023-06-21

**Authors:** Jin-Hui Yin, Wen-Zheng Hu, Yue Huang

**Affiliations:** 1 Beijing Tiantan Hospital, China National Clinical Research Center for Neurological Diseases, Capital Medical University, Beijing, 100070 PR China; 2 Department of Neurology, Beijing Tiantan Hospital, Capital Medical University, Beijing, 100070 PR China; 3 Pharmacology Department, School of Biomedical Sciences, Faculty of Medicine and Health, UNSW Sydney, Sydney, 2032 Australia

**Keywords:** Sandhoff disease, Ataxia, HEXB, Copy number variation (CNV) variant, Hemizygous variation

## Abstract

**Background:**

Sandhoff disease (SD) is a rare neurological disease with high clinical heterogeneity. SD in juvenile form is much rarer and it is often misdiagnosed in clinics. Therein, it is necessary to provide more cases and review the literature on juvenile onset SD.

**Case presentation:**

A 14 years-old boy with eight years of walking difficulties, and was ever misdiagnosed as spinocerebellar ataxia. We found this patient after genetic testing carried rs201580118 and a novel gross deletion in *HEXB* (g.74012742_74052694del). Through review the literature, we found that was the first gross deletion identified at the 3’end of *HEXB*, associated with juvenile onset SD from China.

**Conclusion:**

This case expanded our knowledge about the genotype and phenotype correlations in SD. Comprehensive genetic testing is important for the diagnosis of unexplained ataxia.

**Supplementary Information:**

The online version contains supplementary material available at 10.1186/s12883-023-03267-7.

## Introduction

Sandhoff disease (SD, OMIM 268,800) is an autosomal recessive inheritance disease, caused by variations in *HEXB* (OMIM 606,873). SD is one of monosialoganglioside 2 (GM2) gangliosidoses, which belong to lysosomal storage diseases, caused by deficiency in β-hexosaminidase A(HEXA), β-hexosaminidase B(HEXB) or GM2 activator [1, 2]. *HEXB* encodes the β-subunit of β-hexosaminidase (HEX), which involves the formation of the HEXA and HEXB enzymes. Variations in *HXEB* cause lose normal function of both HEXA and HEXB enzymes, and affect the degradation of GM2 ganglioside. Lysosomal accumulation of GM2 ganglioside results in neuronal cytotoxicity and eventually neuronal death. According to the Cure & Action for Tay-Sachs (CATS) Foundation, the prevalence of SD is about 1 in 300,000 [3]. The clinical presentations of SD are highly heterogeneous with earlier onset severer disease progression (Table 1). According to the onset age, it has been classified as infantile SD, juvenile SD, and adult SD [4, 5]. The most common subtype is infantile SD with onset age before 9 months old. The onset age range of juvenile SD is controversial, which can be between 2 and 10 years old [6] or between 1 and 18 years old [5]. In contrast to the most prevalent infantile SD, the juvenile SD is rarely reported.

**Table 1:** Clinical features of SD subtypes [5, 6]

Subtypes of SD	Onset age	Clinical features	Neuroimaging
Infantile onset	Before the age of 12 months	Most common subtype, acute onset, psychomotor retardation, seizures, blindness, early death blindness and cherry-red spots in the eyes	Bilateral thalamic and basal ganglia abnormal signals
Juvenile onset	1–18 years old	Gait disturbances, speech problems, incoordination, pyramidal signs, muscle wasting, intellectual impairment, dysphagia, seizures, and proximal or distal weakness	Cerebellar/cerebral atrophy
Adult onset	After 18 years old	Motor neuron disease, ataxia (spinocerebellar), tremor (mainly postural), dystonia, psychiatric symptoms, and neuropathy (sensorimotor or autonomic)	Cerebellar/cerebral atrophy

SD = Sandhoff disease

According to the HGMD database, 121 variations have been identified for *HEXB*, including missense/nonsense, splicing, small deletion and indels, and gross deletions. Here, we reported a juvenile SD patient caused by a novel copy number variation (CNV) along with a known variation (rs201580118) in *HEXB*, which broadened the genotype and phenotype scopes of juvenile SD. This study aims to arouse the awareness of a rare juvenile form of SD in clinical practice.

## Case presentation

A 14-year-old boy presented to our neurogenetics outpatient clinic with his parents in a wheelchair. He barely walked without assistance and complained with eight years of progressive disabilities. He was born through full-term vaginal delivery without birth injury nor neonatal asphyxia. At six years old, he developed tremors in his right hand when he wrote or held food. By the time he was seven years old, his tremor became more pronounced, and he started staggering with penguin-like gait and accompanied by slow reactions. Meanwhile, he began to have speech problems with slurred pronunciations and slow speed while talking. At the age of nine, he developed dysphagia and occasionally coughed when drinking water. At the meantime, his dysarthria and ataxia were getting worse. He needed assistance to be able to walk. He developed painful walking spasms when he was 12 years old. Ataxia and dysarthria became more pronounced with occasional fall while walking. Meanwhile, he became irritable and often cried, according to his guardian. He was easy to sweat at the extremities and developed uracratia during his disease course. The boy was taken to a local hospital and underwent a magnetic resonance imaging (MRI) scan, which showed cerebellar atrophy. He was diagnosed with ataxia syndrome or spinocerebellar ataxia (SCA) and treated with vitamins and neuroprotective drugs but without symptoms improvement. There was no genetic evidence suggesting SCAs at that time. His parents were not consanguineous and both of them had been very healthy.

On physical examination, the boy looked emaciated, and his BMI was 17, which was below normal. His up and low extremities were damp and cold. Neurological examination showed that he had slow horizontal saccades with uneven velocity of eye movements and obvious horizontal gaze nystagmus. He had obvious dysarthria during speech and manifested as “poetic” language. His muscle was atrophic, but muscle strength in all four limbs was 5/5. His muscle tone was increased with limb contracture and brisk tendon reflexes. He had bilateral Babinski signs, and ankle clonus was evident. He was unstable on nose-finger test, heel-shin slide test and fast alternating hand movement test. He had obvious intention tremor and difficulty in balancing while sitting and getting worse while standing.

**Fig. 1:**
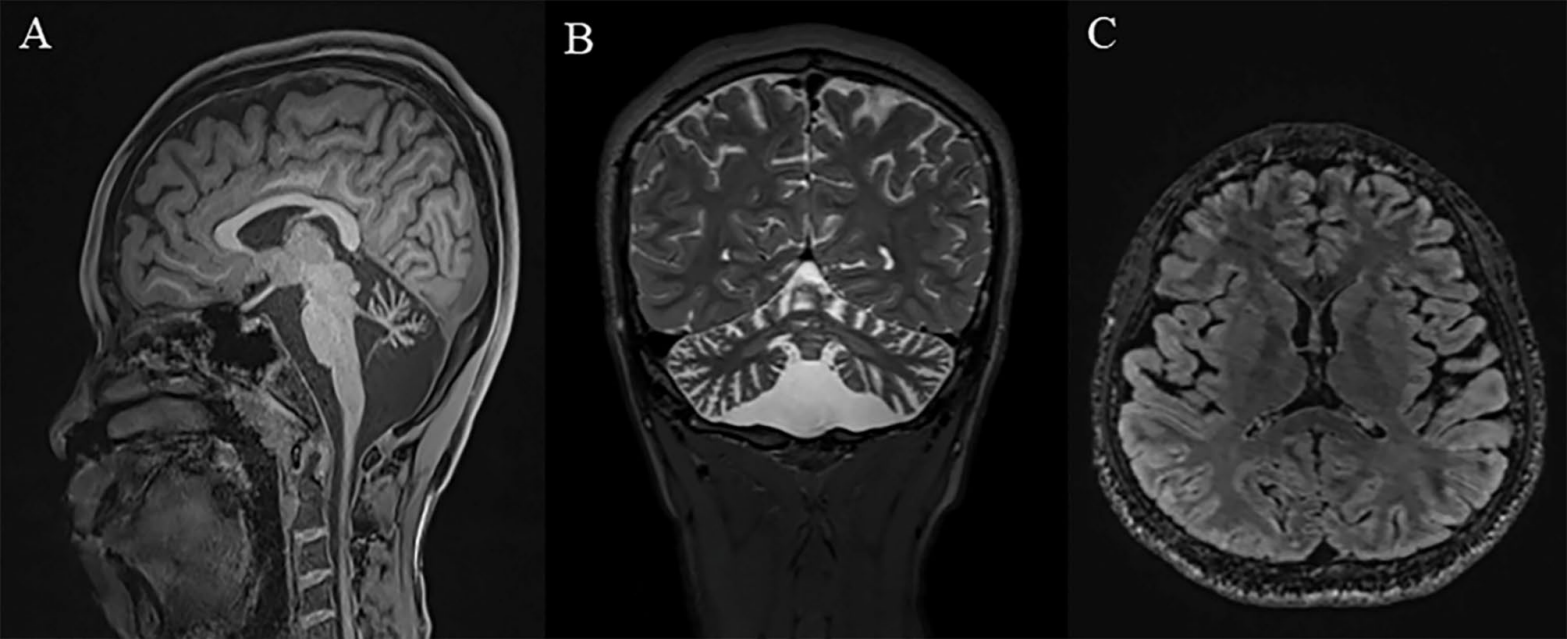
Brain Imaging of this patient. Brain MRI showed severe cerebellar atrophy, enlargement of the fourth ventricle and mild cerebra atrophy. **(A** - T1 image, **B**- T2 image). FLAIR imaging showed the basal ganglia was normal **(C)**

His International Cooperation Ataxia Scale (ICARS) score was 54/100 and Scale for the assessment and rating of ataxia (SARA) was 18.5/49. Psychiatric assessment confirmed a slight depressive state (Beck depression rating scale [BDI] 17/63). His Mini-Mental State Examination (MMSE) score was 18/30. Ophthalmological examination revealed no cherry-red spots in the macula area of the fundus. Abdominal ultrasound examination revealed no enlargement in liver, spleen, or any other organs. Brain MRI showed atrophy in bilateral cerebellar hemispheres, vermis and crus cerebellum. Other regions of the brain were of normal size (Fig. 1).

**Fig. 2:**
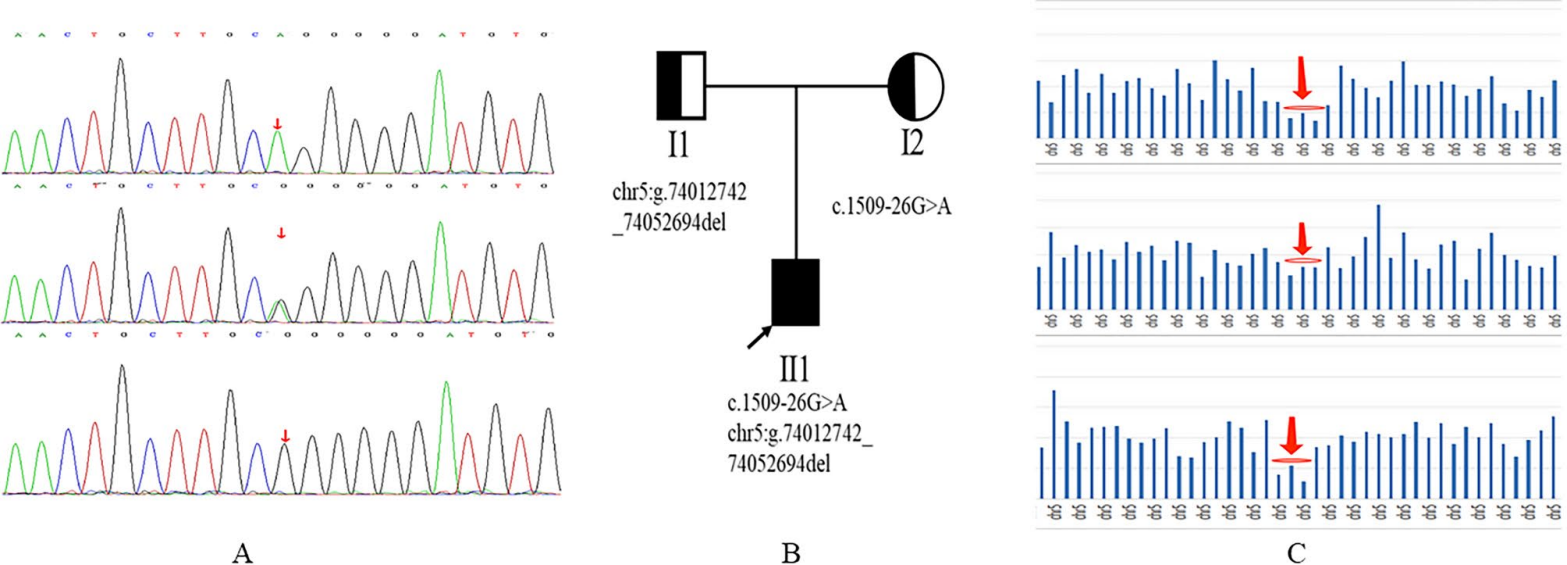
Family tree and gene sequencing data of this patient. From top to bottom were the patient, the patient’s mother, and the patient’s father **(A**, **C)**. Sanger sequencing showed the boy and his mother had c.1509-26G>A variation **(A)**. The genealogical diagram of the family **(B)**. CNV analysis showed the boy had a CNV variant chr5: g. 74012742_74052694del, so did his father. The region indicated by the arrow is the deletion region **(C)**

Based on his presentations and physical examinations, we ordered genetic tests on the patient and his parents. There was no consanguineous marriage in this family (Fig. 2). The whole exome sequencing (WES) data showed that this boy had a known point variation (c.1509-26G > A, rs201580118) and a novel copy number variation (CNV) variant (chr5: g.74012742_74052694del) in *HEXB*. The results were further confirmed by Sanger sequencing and CNV analysis, indicating he inherited the rs201580118 variant from his mother and the CNV variant from his father (Fig. 2). The point variation in *HEXB* c.1509-26G > A was considered as pathogenic/likely pathogenic according to ClinVar database. Another CNV variant chr5: g.74012742_74052694del contained a part of two coding genes *HEXB* and *GFM2*. It covered from the 9th intron of *HXEB* gene to the 5th intron of *GFM2* gene, which was not reported in the Decipher syndrome, ClinVar and gnomAD databases. The patient had a fragment deletion in *HEXB* inherited from his father and a maternal c. 1509-26G > A variation of *HEXB*, which fell within the paternal fragment deletion region of *HEXB*. demonstrating a homozygous form in Sanger sequencing, although it should be attributed to a compound heterozygous variation of *HEXB* in this patient (Fig. 2). To confirm loss function of *HEXB* caused by these variations, we ordered the blood tests to examine leukocytic total β-Hex (HEXA&B) and HEXA activities. Results showed that leukocytic HEXA&B and HEXA actives of this patient were decreased by 92.4% and 21.7% compared to the low limit of normal ranges, which were 75.4-158.6nmol/mg/h and 54.5-140.3nmol/mg/min. The diagnosis of juvenile onset SD was then made. The enzyme activities of HEXA&B of his father and his mother were at 83.5 and 92.2 nmol/mg/h, which were below the median but within the normal range. All family members have consented to publish their clinical data and signed the informed consents.

## Discussion

In this article, we report a juvenile onset SD case with unusual clinical presentations due to compound heterozygous variants in *HXEB*, one of which is a novel large fragmental deletion. This patient had significant gait disturbance, severe ataxia, speech problems, mild cognitive impairment and psychiatric problems, which were the common clinical presentations in juvenile and adult-onset SD [7]. Among them, cerebellar ataxia is the central nervous system symptom mostly observed in juvenile SD (Table 2). Apart from the above five cardinal symptoms, patients could manifest with other varied clinical presentations, ranging from clinical signs indicating involvement of central nervous system, peripheral nervous system, to non-neurological manifestations [5, 6].

**Table 2:** Clinical phenotypes and genotypes comparison of this case with previously reported juvenile onset SD

	Zhang et al. [17]	Maegawa et al. [5]	Moriei et al. [6]	Mugnaini et al. [18]	Wortmann et al. [19]	Zampieri et al. [20]	Gort et al. [21]	Wang et al. [22]	This case
Number of cases	1	6	1	1	1	1	3	6	1
Age at onset(y/o)	6.0	4.8 ± 4.4	10.0	2.0	3.5	1.5	NA	NA	6.0
*HEXB* Genotypes *	c.1509-26G > A c.1404delT	c.1514G>A; c.410G>A c.796T>G c.1057G>C	c.1378T > C c.1598G > A	c.796T > G c.1615 C > T	Homozygous c.851G > A	c.626 C > T c.299 + 1471-408del2406	c.1514G > A c.1250 C > T c.1509-26G > A c.508 C>T	homozygous c.1376 A>C	c.1509-26G > A g.74012742-74052694del
Ethnics	Chinese	American	Japanese	Argentinean	Turkish	Italian	Spanish	Pakistani	Chinese
Gait disturbances	Yes	Yes	Yes	Yes	Yes	Yes	Yes	Yes	Yes
Speech problems	Yes	Yes	Yes	Yes	Yes	Yes	Yes	Yes	Yes
Ataxia	Yes	Yes	No	Yes	Yes	Yes	Yes	Yes	Yes
Cognitive impairment	Yes	Yes	No	Yes	Yes	Yes	NA	Yes	Yes
Psychiatric problems	Yes	Yes	No	Yes	Yes	Yes	Yes	No	Yes
Pyramidal signs	No	Yes	No	No	No	No	Yes	No	Yes
Dysphagia	Yes	Yes	No	Yes	Yes	Yes	NA	Yes	Yes
Muscle wasting	No	Yes	Yes	No	Yes	No	NA	No	Yes
Weakness	No	Yes	Yes	No	Yes	No	NA	Yes	No
Tremor	Yes	Yes	No	No	No	No	NA	Yes	Yes
Developmental delay	No	Yes	No	Yes	Yes	Yes	Yes	No	No
Autonomic dysfunction	Yes	Yes	No	No	No	No	Yes	No	Yes
Tendon hyperreflexia	No	No	Yes	No	No	No	NA	No	Yes
Hypertonia	No	No	No	No	No	No	NA	No	Yes
Hypotonic	No	No	Yes	Yes	No	Yes	Yes	No	No
Limb contracture	No	Yes	Yes	No	No	No	NA	No	Yes
Seizures	No	Yes	No	No	Yes	No	NA	No	No
Paresthesia	No	Yes	Yes	No	No	No	No	No	No
Others	No	Sleep and visual problem	PN; MND- like	IAL	No	CRS; Startle reaction	No	No	Ankle clonus Hyperhidrosis
Neuroimaging									
Cerebellar atrophy	Moderate	Yes^	No	No	NA	NA	Severe(one)	NA	Severe
White matter changes	No	Yes^	No	No	NA	NA	No	NA	No
Residual enzyme activity#									
HEXA&B	3.4–19.8%	1.5–9.9%	10.0–13.0%	2.65%	5.4–12.5%	2.8%	2.9–8.3%	<5%	3.6–7.6%
HEXA	19.0-46.3%	16.7	23.0–27.0%	NA	15.1–41.6%	NA	5.1–7.8%	NA	22.7–58.3%

SD = Sandhoff disease; y/o = years old; NA = Not available; PN = peripheral neuropathy; IAL = infantile autism like; CRS = Cherry-red spot

* NM_000521.4(HEXB) or GRCh37; # The range compared with the upper and lower limits, respectively; ^ represents no imaging available for analysis

This patient was hypertonic and he often had painful spasms while walking due to a significant increase in muscle tone in the lower limbs. Actually, muscle tone in juvenile type SD patients could be both increased [8] or decreased [6], and dystonia could be seen in some juvenile SD patients (Table 2). This patient had pyramidal signs, exaggerated tendon reflexes, and ankle clonus. Indeed, pyramidal signs and increased tendon reflex were only present in a part of juvenile SD patients (Table 2). Individuals with motor neuron disease phenotype of SD had brisk or exaggerated tendon reflexes especially in adults [6, 7]. Those signs indicated upper motor neuron involvement in this patient. Epilepsy could be seen in juvenile SD as well, although it is less common compared to infantile SD [6]. However, epilepsy was not found in this patient. This patient had proximal lower weakness, muscle atrophy, which often seen in juvenile and adult SD. However, due to poor cooperation of this patient, we were not able to perform electromyography to determine whether the patient had changes in cortical spinal neurons.

Although peripheral nerve symptoms were reported in juvenile SD, including paresthesia, peripheral neuropathy (Table 2), we did not identify paresthesia in this patient after a systematic neurological examination. This patient had significant hyperhidrosis and uracratia, indicating autonomic dysfunction. Autonomic dysfunction and peripheral neurology were the main clinical manifestations of adult SD patients in the literature [9, 10]. The main manifestations of autonomic dysfunction in juvenile SD were gastrointestinal dysfunction and uracratia [4]. Arrhythmias, orthostatic hypotension, and abnormal sweating were less commonly reported (Table 2). This patient was thin and emaciate, which was common no-neurological manifestations in SD [5]. There was no any other non-neurological manifestation in this case, although hepatosplenomegaly and fundus lesions, such as cherry-red spot, were the common non-neurological manifestations in almost all infantile SD and some juvenile SD [9] (Table 2).

The brain MRI in this patient showed marked atrophy in bilateral cerebellar hemispheres and mild atrophy in cerebrum, similar to other juvenile SD cases (Table 2). The widespread cortical and subcortical involvement in this case may reflect variable neurodegeneration caused by ganglioside deposition. Interestingly, the neuroimaging features of juvenile SD were brain atrophy, similar to adult SD, but inconsistent with infant SD, which demonstrated abnormal signals in bilateral thalamic and basal ganglia [5, 6] (Tables 1 and 2).

In most juvenile SD cases reported previously, there were no genetic information, and the diagnoses were made based on HEXA&B activity assays only [5, 6]. The β-hexosaminidase (HEX) consists of two major isoenzymes: HEXA, a heterodimer of α and β subunits, and HEXB, a homodimer of β subunits [10]. The variations in *HEXB*, encoding the β subunits present in both isoenzymes, result in deficiency of HEXA and HEXB. The HEXA and HEXA&B activity assays are one of the diagnostic methods for SD. The residual enzyme activities varied among the different types. Lower residual enzyme activity may represent more GM2 ganglioside deposition, more pronounced symptoms, and earlier onset of the disease. There are some residual HEXA&B activities in juvenile and adult SD [11, 12], but absent to near-absent in infantile SD patients [13]. Thus, residual enzymes activities could be a biomarker for disease progression and therapeutic effects evaluation for SD. However, the norms range of the residual enzyme activities varies in different laboratories due to different techniques and different samples used for the test (Table 2). This case report warranted standardization of residual HEX A and HEXA&B activities assay and investigation of other genes that might regulate the levels of residual enzyme activities beyond *HEXB*, which may provide additional therapeutic options for SD.

This patient carried a point variation of rs201580118 and a novel CNV variant (chr5: g.74012742_74052694del). This about 40Kb deletion starts in the 9th intron of *HXEB*, covering exons 10–14 of *HEXB* and a part of *GFM2*. This gross deletion variation is the first reported in the 3’end of *HEXB*. In contrast, 16 Kb and 50Kb gross deletions at the 5’ end of *HEXB* have been reported in different other ethnics. They are located at the 5’end, spanning the promoter, exons 1–5 and part of intron 5 [14], and along with other variations, their correspondent clinical phenotype is infantile SD [15]. There are various variations causing adult or juvenile onset SD [16, 17]. This 40Kb deletion in our case extended the gross deletion forms of *HEXB*, and along with rs201580118, composed a hemizygous variation in *HEXB*, caused loss function of HEXA&B and HEXA, and corresponded to juvenile onset SD (Table 2).

### Limitation of this study

HEXA&B are two related enzymes and each of these enzymes is made up of two subunits. HEXA includes one alpha subunit (produced from HEXA) and one beta subunit (produced from HEXB), while HEXB is composed of two beta subunits. In this study, we only could measure total enzyme activities of HEXA&B in all family members and HEXA in this patient to provide indirect evidence showing function of HEXB variants. In addition, longitudinal observation is required to testify whether the onset age and disease severity are related to the enzyme activities of HEXA&B.

In this study, we described a juvenile SD patient, and his diagnosis was made by the evidences from HEXA&B enzyme assays, genetic analysis, clinical features, and neuroimaging. This patient carries a novel approximate 40Kb deletion and a known pathogenic point variant in *HEXB*. He has several clinical manifestations that are less common in adolescent SD patients. Our case report expands the genetic variation spectrum leading to SD. Although SD is a very rare disease, particularly in its juvenile and adult forms, this case report suggests that when we encounter a patient with ataxia, the possibility of SD should be considered.

## Electronic supplementary material

Below is the link to the electronic supplementary material.

**Supplementary Figure.** Comparison of copy number histogram in the region chr5:72914062-75031518. The copy number histograms from the top to the bottom are from the patient, his mother, and his father. The arrow indicates the region with fragment deletion

## Data Availability

Access to the full data used in the study is available from the authors upon request.

## Abbreviations

SD: Sandhoff disease
GM2: Monosialoganglioside 2
HEXA: β-hexosaminidase A
HEXB: β-hexosaminidase B
HEX: β-hexosaminidase
MRI: Magnetic resonance imaging
SCA: Spinocerebellar ataxia
ICARS: International Cooperation Ataxia Scale
SARA: Scale for the assessment and rating of ataxia
BDI: Beck depression rating scale
MMSE: Mini-Mental State Examination
WES: Whole exome sequencing
CNV: Copy number variation
HEXA&B: Total β-Hex.
