# Supplementary figures and images for: Clinical and genetic features of a case with juvenile onset sandhoff disease
Source: BMC Neurol. 2023 Jun 21;23:240. doi: 10.1186/s12883-023-03267-7 (PMC10286434; doi:10.1186/s12883-023-03267-7)

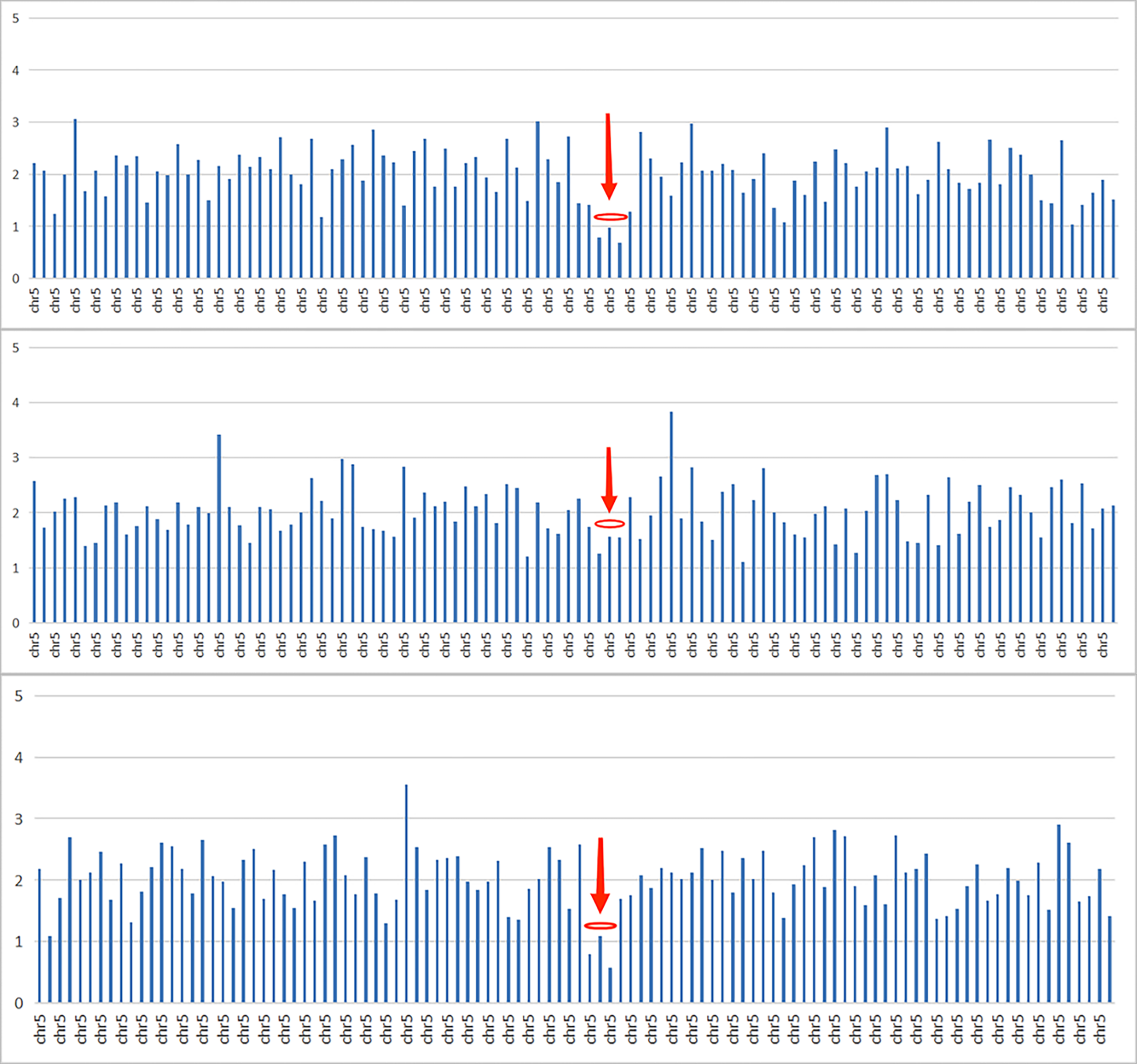

Supplement: Supplementary file 1 — Supplementary Figure. Comparison of copy number histogram in the region chr5:72914062-75031518. The copy number histograms from the top to the bottom are from the patient, his mother, and his father. The arrow indicates the region with fragment deletion [file 12883_2023_3267_MOESM1_ESM.tif]
